# Loss of long-chain acyl-CoA dehydrogenase protects against acute kidney injury

**DOI:** 10.1172/jci.insight.186073

**Published:** 2025-02-11

**Authors:** Takuto Chiba, Akira Oda, Yuxun Zhang, Katherine Pfister, Joanna Bons, Sivakama S. Bharathi, Ayako Kinoshita, Bob B. Zhang, Adam Richert, Birgit Schilling, Eric Goetzman, Sunder Sims-Lucas

**Affiliations:** 1 Department of Pediatrics, University of Pittsburgh Medical Center Children’s Hospital of Pittsburgh (UPMC CHP), University of Pittsburgh, Pittsburgh, Pennsylvania, USA.; 2 Buck Institute for Research on Aging, Novato, California, USA.

**Keywords:** Metabolism, Nephrology, Cell stress, Fatty acid oxidation

## Abstract

The renal tubular epithelial cells (RTECs) are particularly vulnerable to acute kidney injury (AKI). While fatty acids are the preferred energy source for RTECs via fatty acid oxidation (FAO), FAO-mediated H_2_O_2_ production in mitochondria has been shown to be a major source of oxidative stress. We have previously shown that a mitochondrial flavoprotein, long-chain acyl-CoA dehydrogenase (LCAD), which catalyzes a key step in mitochondrial FAO, directly produces H_2_O_2_ in vitro. Furthermore, we showed that renal LCAD becomes hyposuccinylated during AKI. Here, we demonstrated that succinylation of recombinant LCAD protein suppresses the production of H_2_O_2_. Following 2 distinct models of AKI, cisplatin treatment or renal ischemia/reperfusion injury (IRI), *LCAD^–/–^* mice demonstrated renoprotection. Specifically, *LCAD^–/–^* kidneys displayed mitigated renal tubular injury, decreased oxidative stress, preserved mitochondrial function, enhanced peroxisomal FAO, and decreased ferroptotic cell death. LCAD deficiency confers protection against 2 distinct models of AKI. This suggests a therapeutically attractive mechanism whereby preserved mitochondrial respiration as well as enhanced peroxisomal FAO by loss of LCAD mediates renoprotection against AKI.

## Abstract 2

Renal TECs are prone to AKI due to FAO-related oxidative stress. LCAD deficiency protects against AKI by reducing injury and stress, preserving function, and enhancing peroxisomal FAO.

## Graphical abstract

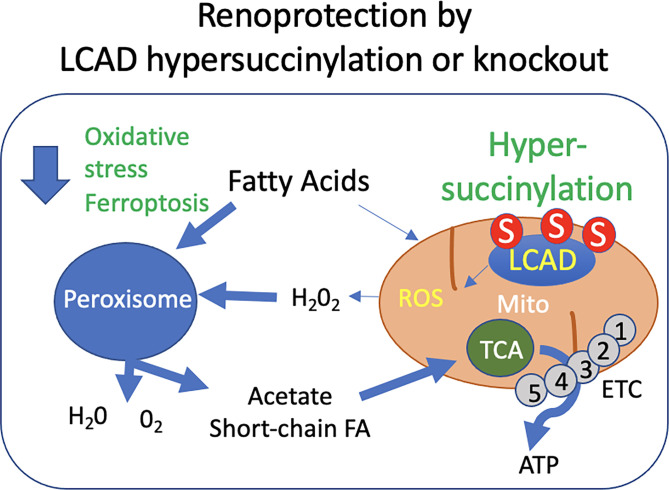

## Introduction

Dysregulated energy metabolism has long been recognized as a fundamental element of acute kidney injury (AKI), yet no effective therapeutic modalities have been developed to specifically target renal metabolic dysfunction (1). Mitochondrial fatty acid oxidation (FAO), the process by which carbon chains of fatty acids are shortened for energy production, has been implicated as a source of H_2_O_2_ and oxidative stress in various tissues including kidneys (2, 3). Tubular epithelial cells (TECs), which are the main site of damage during AKI, are rich in mitochondria and rely on FAO for their high energy demands (4, 5). Previous studies in AKI have shown that mitochondrial reactive oxygen species (ROS) build up is a pivotal driver of cellular injury and cell death (6–8).

Long-chain acyl-CoA dehydrogenase (LCAD) catalyzes the first step in mitochondrial FAO. Mitochondrial FAO is integrated with the electron transport chain that produces ROS (9). We recently showed that LCAD in the presence of fatty acids can lead to the direct production of H_2_O_2_ as a biproduct in vitro (10). In addition, LCAD is downregulated in many cancers, possibly as a mechanism to reduce H_2_O_2_ levels (11, 12). Lysine acylation is a known posttranslational modification to inhibit LCAD activity (13, 14). It is not clear how LCAD is regulated during AKI or how it may contribute to tissue injury or recovery.

Here, we show via mass spectrometry data analysis that AKI induces LCAD hyposuccinylation in the kidney. In vitro assays with recombinant LCAD protein suggest that this hyposuccinylation may increase LCAD-generated H_2_O_2_, thereby contributing to tissue damage during AKI. Indeed, our studies demonstrate that loss of LCAD in mice confers renoprotection against cisplatin and renal IRI, which are 2 distinct mouse models of AKI. Renoprotection after AKI involves preservation of mitochondrial respiration and enhanced peroxisomal FAO. *LCAD^–/–^* mitigates oxidative stress and ferroptosis (oxidative stress induced cell death) after AKI. These results provide evidence for a potential therapeutic approach for the treatment of AKI.

## Results

### LCAD hypersuccinylation is associated with renoprotection after AKI.

**Figure 1:**
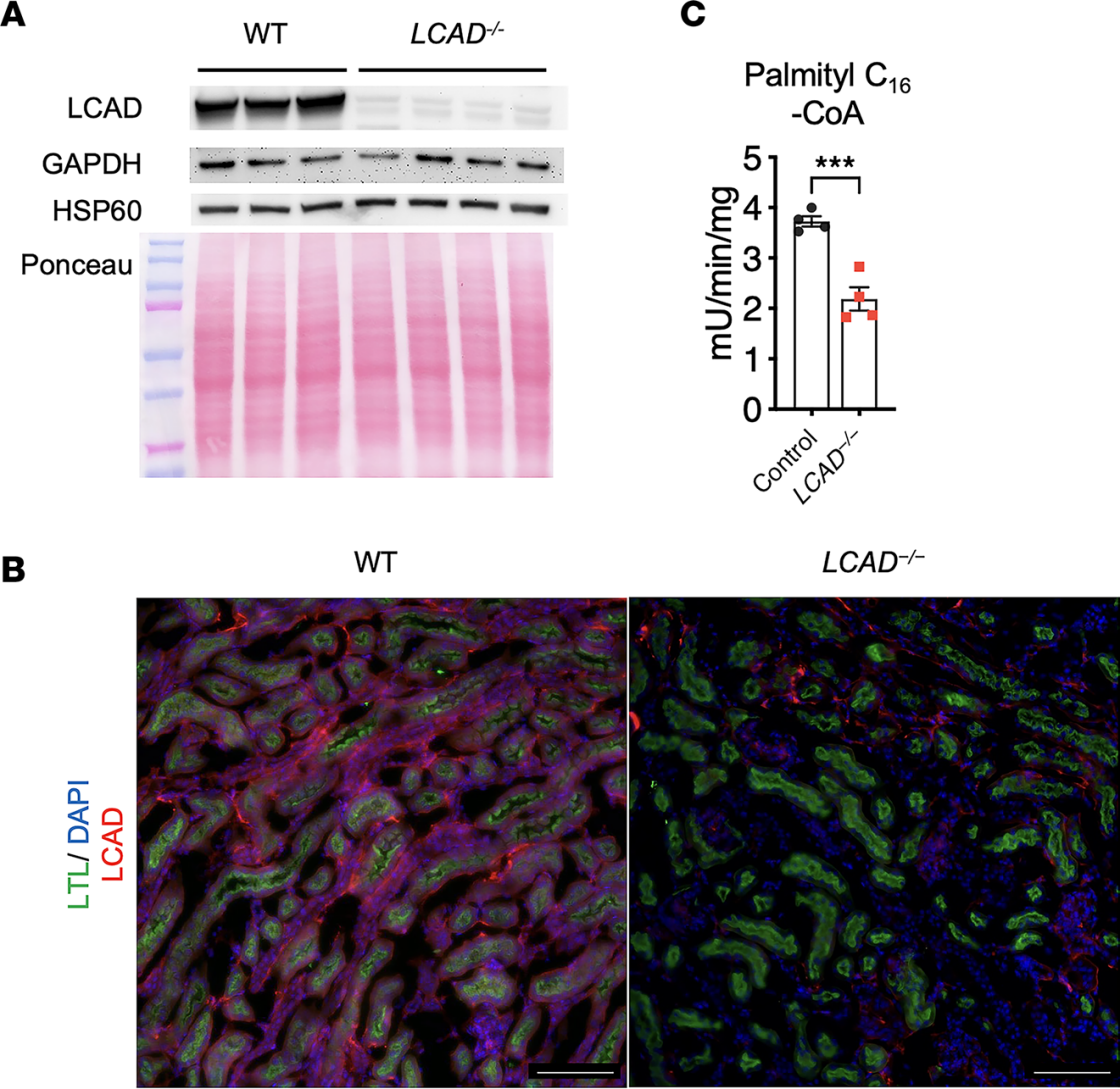
LCAD expression in the kidneys. (**A**) Anti-LCAD immunoblot in *LCAD^–/–^* versus WT mouse kidney lysates. GAPDH, loading control; HSP60, mitochondrial loading control. (**B**) Anti-LCAD immunostaining in *LCAD^–/–^* versus WT mouse kidney, costained with Lotus Tetragonolobus Lectin (LTL). (**C**) *LCAD^–/–^* versus WT mouse kidney homogenates were tested for palmitoyl-CoA (C_16_-CoA) dehydrogenase activities. *n* = 3–4. Scale bars: 100 μm. ***P < 0.001, using *t* test.

To study the role of LCAD in AKI, we generated a line of LCAD-KO mice. We confirmed that WT mice express LCAD in the kidney including LTL^+^ proximal TECs, whereas *LCAD^–/–^* kidneys do not (Figure 1, A and B). Electron transfer flavoprotein (ETF) fluorescence reduction assay was used to measure acyl-CoA dehydrogenase enzyme activity. Mitochondria isolated from WT or *LCAD^–/–^* kidneys were compared using palmitoyl (C_16_)-CoA as a substrate, representing the combined activities of LCAD, very LCAD (VLCAD), and ACAD9 (mostly LCAD and VLCAD). Interestingly, *LCAD^–/–^* kidney mitochondria show only about 40% reduction of combined acyl-CoA dehydrogenase activity with C_16_-CoA (Figure 1C), whereas an approximately 65% reduction was reported in *LCAD^–/–^* liver mitochondria (15). This suggests that, in kidneys, VLCAD plays a substantial role in mitochondrial long-chain FAO.

**Figure 2:**
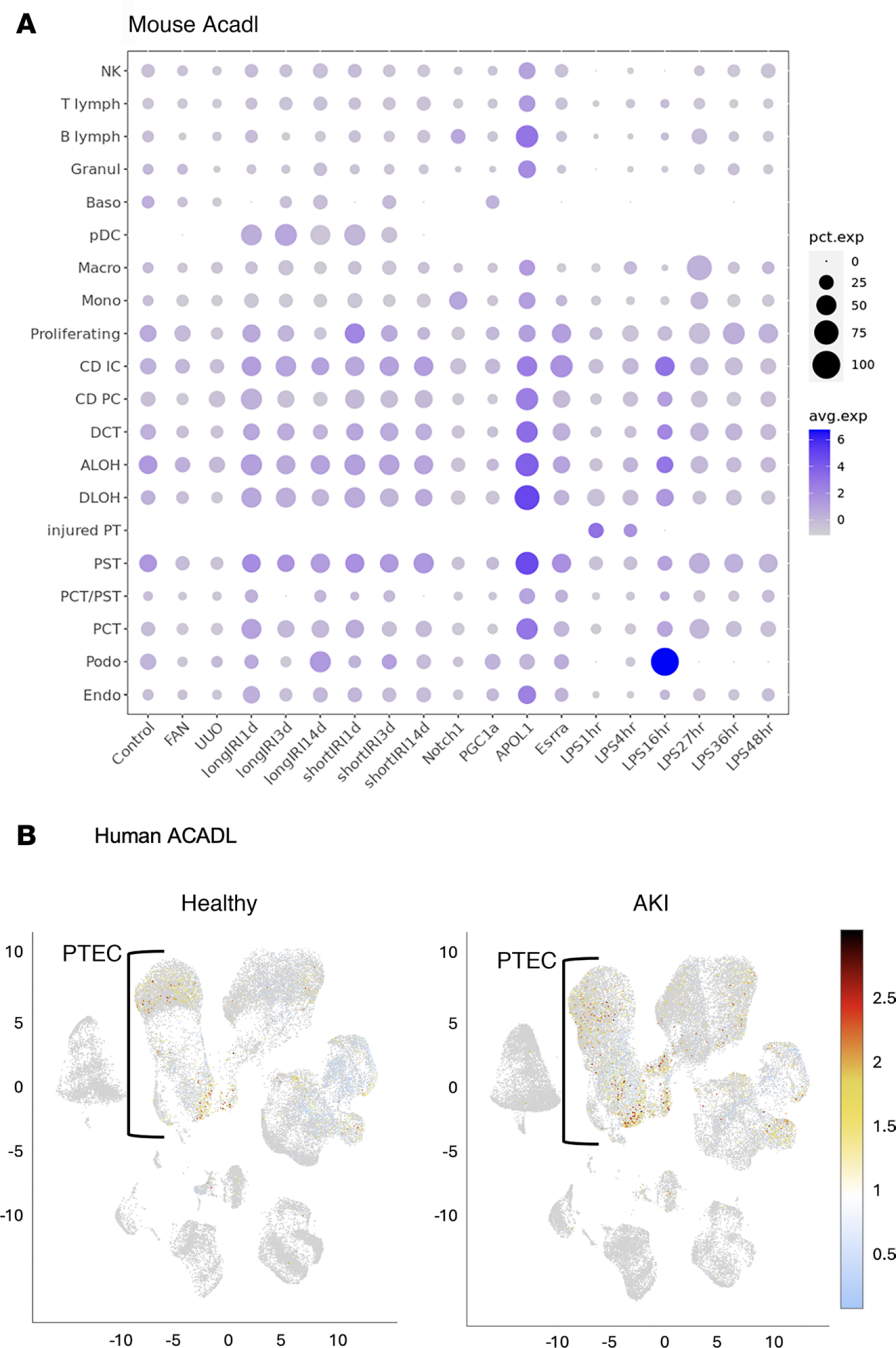
Differential expression of *ACADL* (LCAD) in renal cell clusters from healthy and AKI mouse and human kidneys. (**A**) Data were derived from the Mouse Kidney scRNA-Seq Atlas from Katalin Susztak’s laboratory (https://susztaklab.com). scRNA-Seq data (36 samples) and bulk gene expression data (42 samples) from 18 commonly used mouse kidney disease models. (**A**) Mouse kidney models used to generate the scRNA-Seq atlas: APOL1, *Apol1* transgenic; Control, WT control; Esrra, *Esrra* KO; FAN, folic acid nephropathy; IRI, ischemia reperfusion injury; LPS, endotoxin injection; Notch1, *Notch1* transgenic; PGC1α, *Pgc1a* transgenic; UUO, unilateral ureteral obstruction. Note, IRI contains long and short IRI samples collected 1, 3, and 14 days after the ischemia; LPS contains samples obtained at 1, 4, 16, 27, 36, and 48 hours after the LPS injection. (**B**) Data were derived from the Kidney Precision Medicine Project (KPMP) Kidney Tissue Atlas. Healthy, *n* = 28; AKI, *n* = 14; CKD, *n* = 37. Data were derived from the Kidney Precision Medicine Project (KPMP) Kidney Tissue Atlas, which was accessed on November 1, 2024.

**Figure 3:**
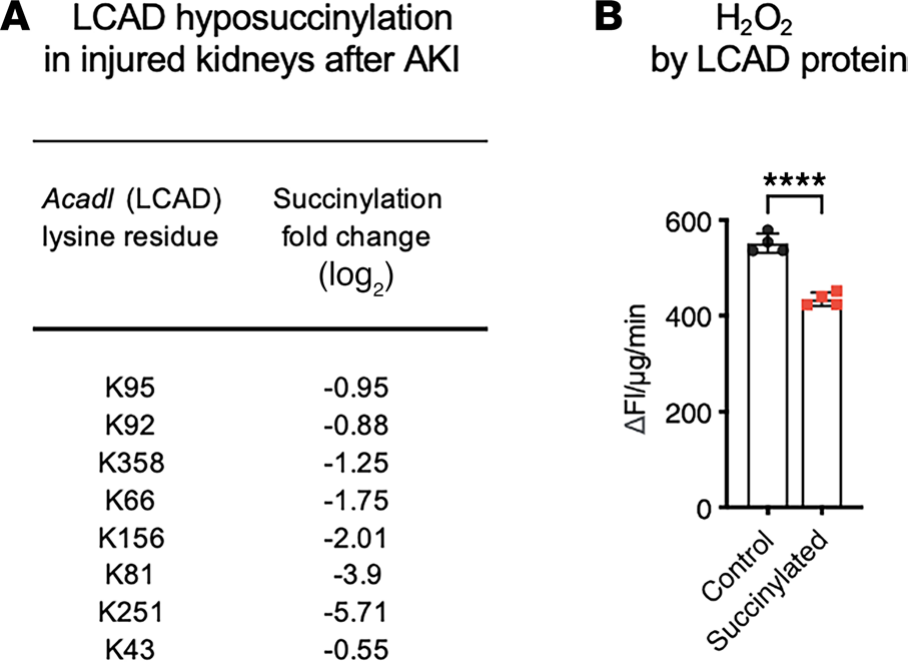
LCAD succinylation linked to renoprotection. (**A**) Mass spectrometry revealed hyposuccinylation of several lysine residues of LCAD in kidneys after AKI compared with contralateral uninjured kidneys. (**B**) Succinylation reduces mouse LCAD oxidase activity. *n* = 4, ****P < 0.0001, using *t* test.

Cell type–specific expression of *ACADL* (LCAD) in the kidney was then explored using publicly available single cell RNA-Seq (scRNA-Seq) data for human and mouse kidney. LCAD was found to be highly expressed in injured TEC in both human and mouse kidney (Figure 2, Supplemental Figure 1, and Supplemental Table 1; supplemental material available online with this article; https://doi.org/10.1172/jci.insight.186073DS1). Notably, LCAD is highly expressed in the S3 region of the proximal tubules in healthy mouse kidneys, especially in males (Supplemental Figure 2). Furthermore, a targeted analysis of LCAD lysine succinylation in our previously published dataset revealed that several LCAD lysine residues are hyposuccinylated in injured kidneys after AKI (Figure 3A) (16). To determine how succinylation status of the LCAD protein may be linked to injury, we performed an in vitro assay with recombinant human LCAD that was chemically succinylated with succinyl-CoA. Succinylation of the LCAD protein was shown to reduce H_2_O_2_ production (Figure 3B), suggesting a mechanism in which hyposuccinylation of LCAD during AKI increase oxidative stress.

We have previously shown that loss of the lysine deacylase sirtuin 5 confers renoprotection against AKI. We have shown that mitochondrial FAO enzymes are hypersuccinylated to inhibit their activity in *Sirt5^–/–^* kidneys. Metabolic adaptation to blocked mitochondrial FAO involves compensatory FAO in the peroxisome, resulting in mitigation of oxidative stress and renoprotection (17). We now show that succinylation of LCAD residue K322 is increased by 16-fold in *Sirt5^–/–^* kidney after AKI (Supplemental Figure 3). Succinylation of residue K322, which is near the LCAD active site, suppresses enzymatic activity (14). However, chemical succinylation of a mutant recombinant LCAD protein containing a conservative substitution at residue 322 (Lys to Arg) that cannot be acylated, still suppressed H_2_O_2_ formation, indicating that the effect of lysine succinylation on LCAD H_2_O_2_ production is mediated by other lysine residues (Supplemental Figure 4). Taken together, our data suggest that LCAD succinylation is broadly associated with renoprotection after AKI. Because LCAD can be a source of mitochondrial H_2_O_2_, we hypothesized that the loss of function of LCAD attenuates oxidative stress to protect against AKI (10).

### LCAD^–/–^ kidneys are protected against distinct AKI models by cisplatin or renal IRI in mice.

**Figure 4:**
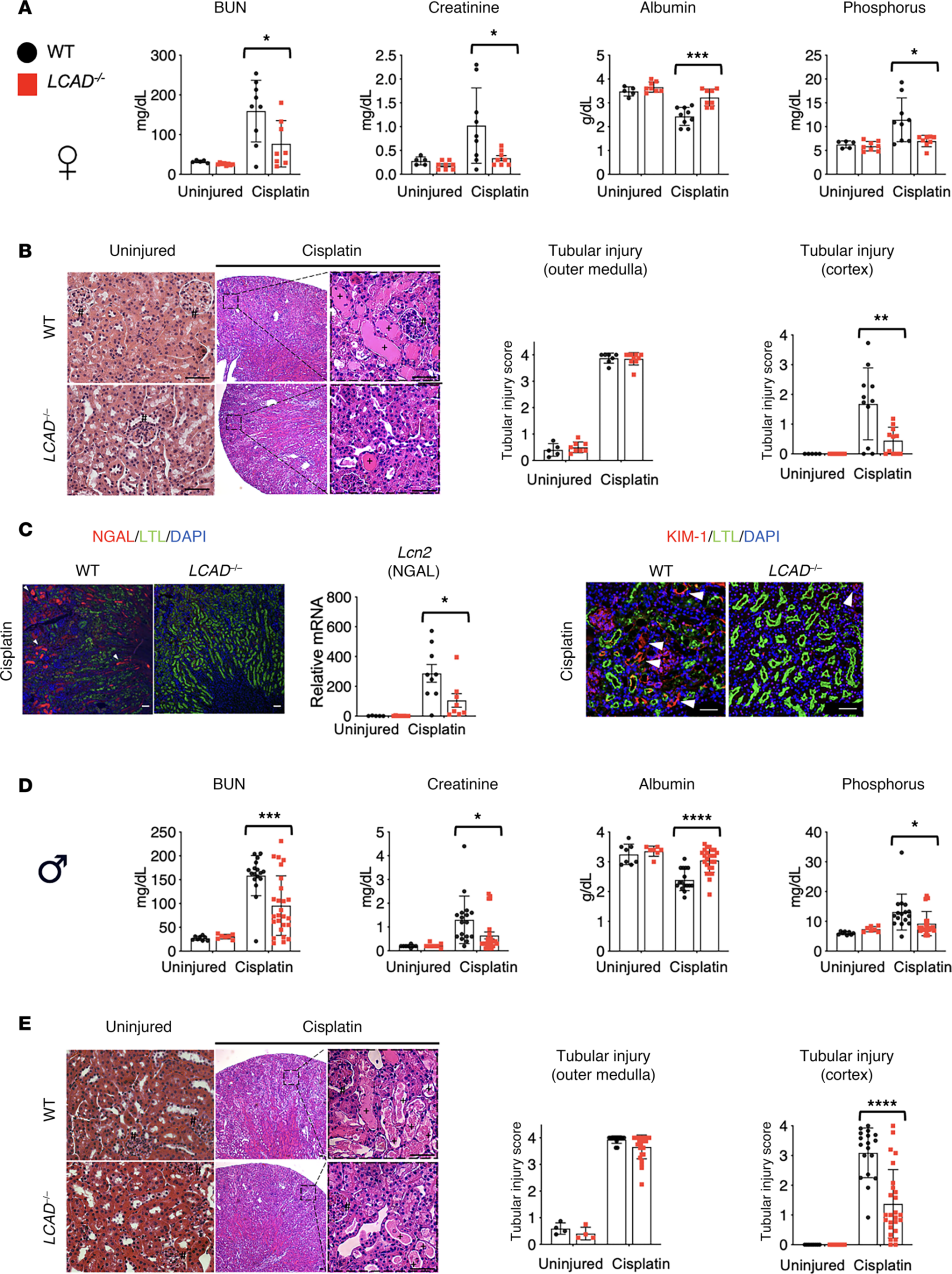
*LCAD^–/–^* kidneys are protective against cisplatin-AKI in sex-independent manner. (**A**–**C**) Female mouse studies. (**D** and **E**) Male studies (**A** and **D**) Serum analyses for BUN, creatinine, Albumin and Phosphorus indicate that the level of renal functional impairment is decreased in *LCAD^–/–^* mice 3 days post cisplatin treatment in sex independent manner. (**B** and **E**) H&E-stained kidney tissues demonstrate that renal tubular injury is mitigated in *LCAD^–/–^* kidneys 3 days after cisplatin treatment in sex independent manner. Glomeruli indicated by #; proteinaceous cast indicated by +. *n* = 8–11. Representative images and semiquantitative scoring for renal tubular damage at day 3 after cisplatin treatment are shown. Cortex and outer medulla regions are scored separately. (**C**) *LCAD^–/–^* kidneys decreased mRNA (*Lcn2*) and immunostained tubular cells for NGAL or KIM-1 in whole kidneys 3 days after cisplatin treatment. Scale bars: 50 μm. *P < 0.05; ***P < 0.001; ****P < 0.0001, using 1-way ANOVA post hoc Tukey multiple comparison. Arrowheads indicate damaged tubules.

We confirmed that no difference was noted in renal function and renal histology in the absence of injury between *LCAD^–/–^* and WT kidneys (Figure 4, uninjured). We next used a single high dose of cisplatin (20 mg/kg b.w., i.p.) to induce nephrotoxic AKI on WT or *LCAD^–/–^* female mice. *LCAD^–/–^* mice had decreased renal injury 3 days after cisplatin, assessed by decreased serum levels of BUN, creatinine, and phosphorus, with an attenuated reduction in serum albumin (Figure 4A). Decreased serum albumin was reported in patients treated with cisplatin (18, 19). Serum albumin decline in patients who developed AKI is associated with mortality (20). We have previously observed that serum albumin is significantly elevated in *LCAD^–/–^* lavage fluid (21). Histology and immunostaining further confirmed reduced renal tubular injury in renal cortex area as well as reduced expression of kidney injury markers of NGAL and KIM1 in *LCAD^–/–^* kidney tissue (Figure 4, B and C). We also analyzed the mRNA levels of inflammatory macrophage markers. Interestingly, in the cisplatin groups, the macrophage markers were downregulated to undetectable levels in most samples after cisplatin treatment, regardless of genotype (Supplemental Figure 5). It appears that bone marrow suppression was induced with our high dose of cisplatin after its treatment at the day 3 time point, and there was little macrophage infiltration, which is consistent with the literature (22). We also performed the corresponding experiments in male mice and observed consistent results with the females, suggesting that our findings are sex independent (Figure 4, D and E).

**Figure 5:**
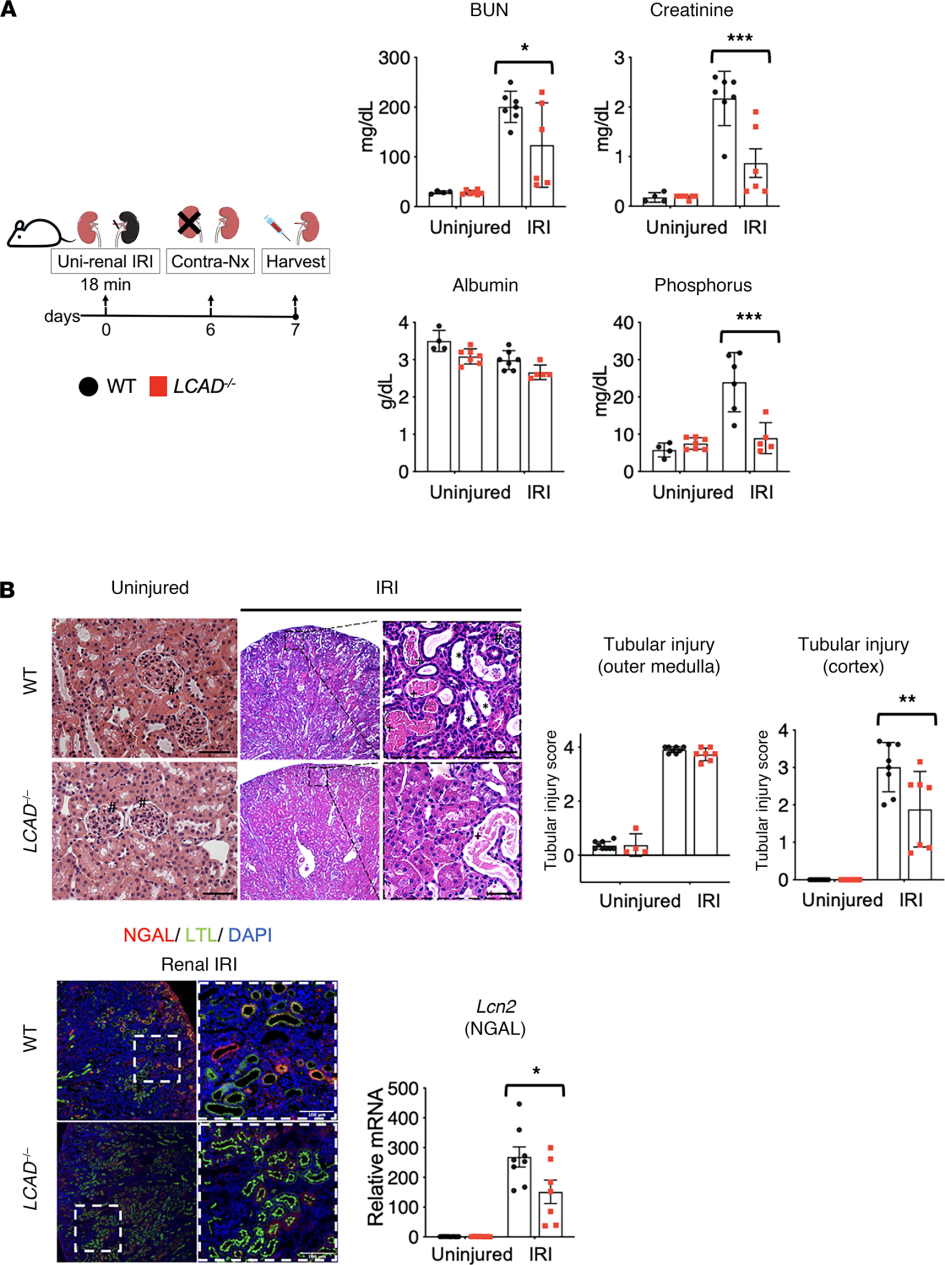
*LCAD^–/–^* kidneys are protective against ischemic-AKI. (**A**) Schematic of ischemia reperfusion injury regime. Serum analyses for BUN, creatinine, and phosphorus indicate that the level of renal functional impairment is decreased in *LCAD^–/–^* mice 7 days after renal IRI in male mice. (**B**) H&E-stained kidney tissues demonstrate that renal tubular injury is mitigated in *LCAD^–/–^* kidneys 7 days postrenal IRI. Glomeruli indicated by #; proteinaceous cast indicated by +; dilated tubule indicated by *. *n* = 6–7. Representative images and semiquantitative scoring for renal tubular damage at day 7 after renal IRI are shown. Cortex and outer medulla are scored separately. *LCAD^–/–^* kidneys decreased mRNA (*Lcn2*) and immunostained tubular cells for NGAL in whole kidneys 7 days after renal IRI. Scale bars: 50 μm. *P < 0.05; **P < 0.01; ***P < 0.001, using 1-way ANOVA post hoc Tukey multiple comparison.

*LCAD^–/–^* and WT male mice were also subjected to an ischemic AKI model in which we used unilateral renal ischemia/reperfusion injury, followed by delayed contralateral nephrectomy 1 day before the samples were collected on day 7 (Figure 5 and Supplemental Figure 6). We obtained consistent results from the cisplatin-AKI model, suggesting that *LCAD^–/–^* kidneys are protective against multiple distinct AKI models.

### Mitochondrial function and peroxisomal FAO are preserved in LCAD^–/–^ kidneys after cisplatin-AKI.

**Figure 6:**
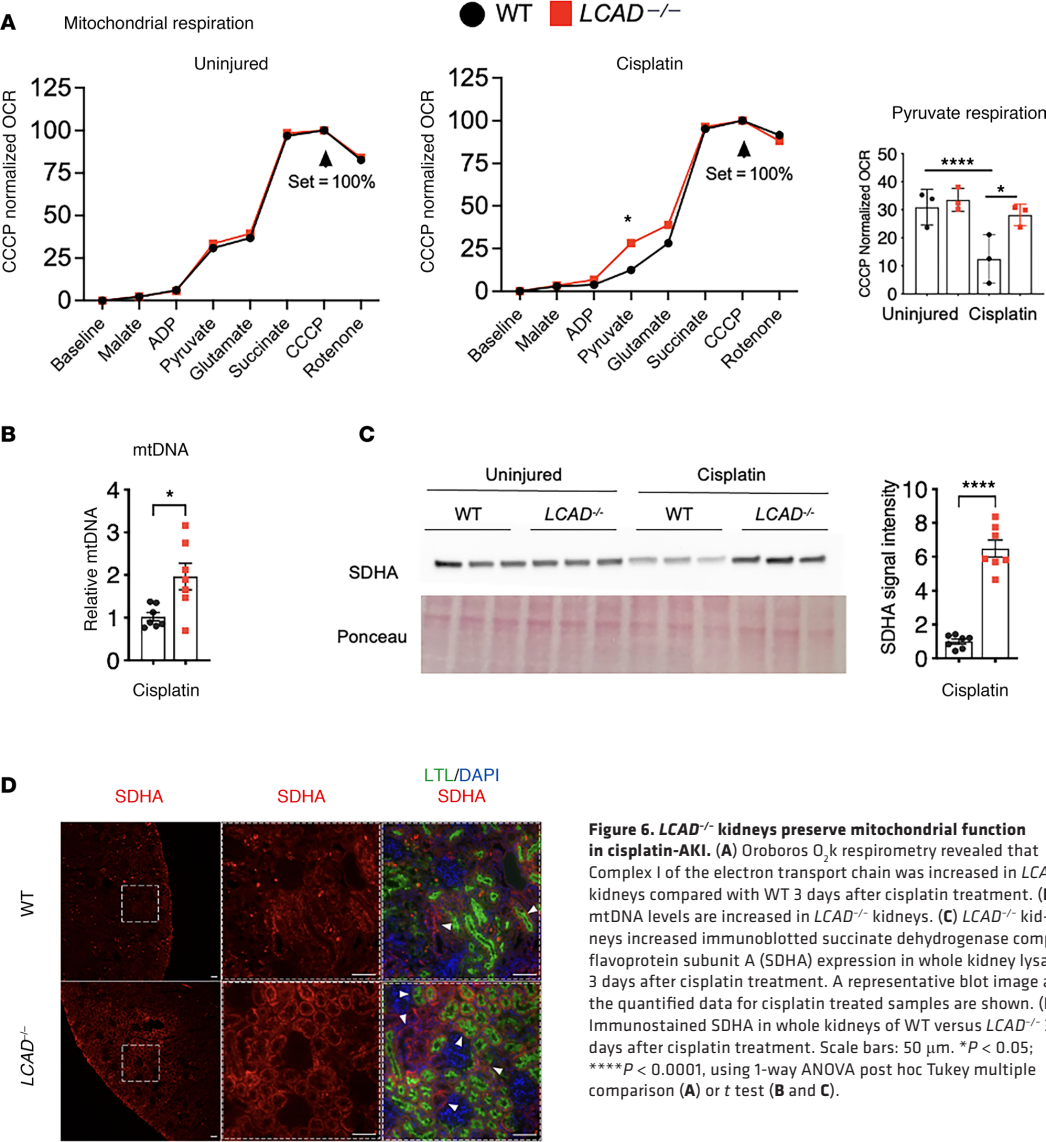
*LCAD^–/–^* kidneys preserve mitochondrial function in cisplatin-AKI. (**A**) Oroboros O_2_k respirometry revealed that Complex I of the electron transport chain was increased in *LCAD^–/–^* kidneys compared with WT 3 days after cisplatin treatment. (**B**) mtDNA levels are increased in *LCAD^–/–^* kidneys. (**C**) *LCAD^–/–^* kidneys increased immunoblotted succinate dehydrogenase complex flavoprotein subunit A (SDHA) expression in whole kidney lysates 3 days after cisplatin treatment. A representative blot image and the quantified data for cisplatin treated samples are shown. (**D**) Immunostained SDHA in whole kidneys of WT versus *LCAD^–/–^* 3 days after cisplatin treatment. Scale bars: 50 μm. *P < 0.05; ****P < 0.0001, using 1-way ANOVA post hoc Tukey multiple comparison (**A**) or *t* test (**B** and **C**).

We analyzed mitochondrial respiration on carbohydrate-derived substrates using the Oroboros Oxygraph-2K high-resolution respirometer with *LCAD^–/–^* or WT kidneys with or without cisplatin-AKI. As in liver, *LCAD^–/–^* mitochondria in uninjured kidneys showed no change in respiratory chain function (10). Interestingly, respiratory chain function is also preserved in injured *LCAD^–/–^* mitochondria, while injured WT mitochondria demonstrated a significant decrease in respiration on the Complex I substrate pyruvate (Figure 6A). Succinate dehydrogenase complex flavoprotein subunit A (SDHA) is a component for SDH, which is a critical mitochondrial enzyme for the TCA cycle and the electron transport chain. Consistent with the Oroboros respirometric data, mitochondrial DNA (mtDNA) level is increased and SDHA expression is preserved in injured *LCAD^–/–^* kidneys (Figure 6, B–D).

**Figure 7:**
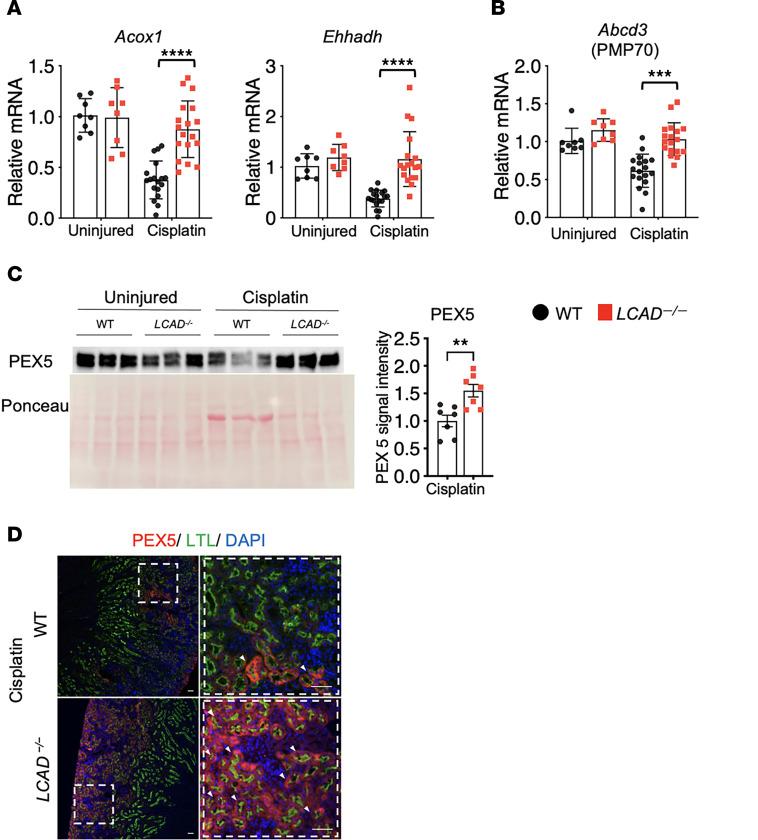
*LCAD^–/–^* kidneys enhance peroxisomal fatty acid oxidation (FAO) in cisplatin-AKI. (**B**) *LCAD^–/–^* kidneys increase mRNA levels of peroxisomal FAO associated genes (*Acox1* and *Ehhadh*). (**B**) mRNA levels of PMP70 (peroxisomal marker) are increased in *LCAD^–/–^* kidneys 3 days after cisplatin treatment. (**C**) Immunoblotted PEX5 (peroxisomal marker) is increased in *LCAD^–/–^* kidneys 3 days after cisplatin treatment. (**D**) PEX5 immunostaining images are also shown. Scale bars: 50μm. *P < 0.05; **P < 0.01; ***P < 0.001; ****P < 0.0001, using 1-way ANOVA post hoc Tukey multiple comparison or *t* test.

We and others have previously shown that enhanced peroxisomal function is linked to renoprotection against AKI (16, 17, 23, 24). We therefore interrogated *LCAD^–/–^* kidneys for peroxisomal FAO after cisplatin AKI. It is consistent that 2 peroxisomal FAO genes (*Acox1* and *Ehhadh*) were upregulated in injured *LCAD^–/–^* kidneys (Figure 7A). *LCAD^–/–^* kidneys were confirmed to have greater abundance of peroxisomal membrane markers PMP70 and PEX5 in the kidneys after cisplatin-AKI, suggesting preservation not only of function but also the number of peroxisomes (Figure 7, B–D).

### Oxidative stress and ferroptotic cell death are decreased in LCAD^–/–^ kidneys after cisplatin-AKI.

**Figure 8:**
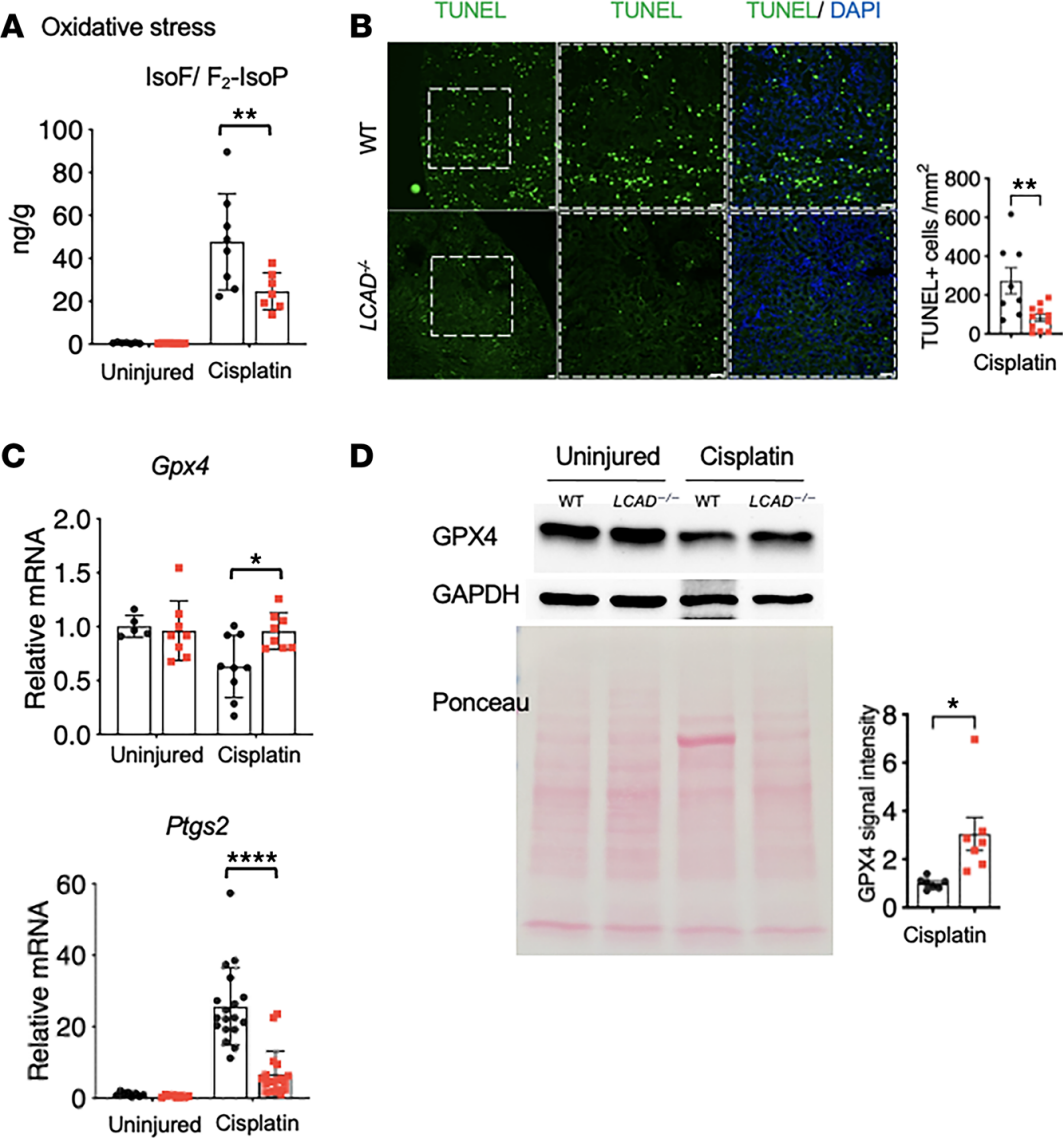
*LCAD^–/–^* kidneys mitigate oxidative stress and ferroptosis in cisplatin-AKI. (**A**) The in vivo oxidative stress marker, isofuran/F_2_- Isoprostane (IsoF/F_2_-IsoP) ratio, is decreased in *LCAD^–/–^* kidneys 3 days after cisplatin treatment. (**B**) TUNEL^+^ dying cells are decreased in *LCAD^–/–^* kidneys 3 days after cisplatin treatment. (**C**) Ferroptosis associated genes *Ptges2* are decreased while *Gpx4* is increased in *LCAD^–/–^* kidneys 3 days after cisplatin treatment. (**D**) Immunoblotted GPX4 expression is increased in *LCAD^–/–^* kidneys 3 days after cisplatin treatment. Scale bars: 50 μm. *P < 0.05; **P < 0.01; ****P < 0.0001, using 1-way ANOVA post hoc Tukey multiple comparison or *t* test.

Consistent with our overarching hypothesis, an in vivo marker of oxidative stress, isofuran/F_2_-isoprostane (IsoPs) (25), was decreased in the *LCAD^–/–^* kidneys after cisplatin AKI (Figure 8A). To further strengthen the mechanism of decreased oxidative stress in the *LCAD^–/–^* kidneys, we performed in vitro analysis on primary isolated proximal tubules in the presence of combined glucose oxygen deprivation. Here we found that proximal tubule cells that had deleted LCAD were protected and had less oxidative stress than WT cells upon CGOG treatment. We observed lower levels of the Mitosox in the *LCAD^–/–^* cells as well as lower levels of thioredoxin reductase (TrxRD) and 4-Hydroxynonenal (4HNE), which are clear indicators of a blunted stress response in these cells (Supplemental Figure 7).

Ferroptosis is a regulated form of cell death by iron-dependent lipid peroxidation in which mitochondrial oxidative stress plays an important role (26). Abnormal fatty acid metabolism is essential for the delivery of the molecular signals to induce ferroptotic cell death (27). We performed TUNEL analysis, a universal assay for programmed cell death including ferroptosis, and showed that *LCAD^–/–^* kidneys had decreased TUNEL^+^ cells after cisplatin AKI (Figure 8B). We further tested 3 ferroptosis markers Gpx4 (downregulated in ferroptosis), Alox5, and Ptges2 (both upregulated in ferroptosis). We have shown that *LCAD^–/–^* kidneys have increased levels of Gpx4 and decreased levels of Alox5 and Ptges2, which suggests reduced ferroptosis (Figure 8, C and D).

## Discussion

Renal TECs (RTECs), which are the major site of injury during AKI, are rich in mitochondria and rely on FAO for their high energy demands (4, 5). During AKI, mitochondria are damaged, FAO is compromised, and toxic lipids accumulate, thereby leading to oxidative stress (3, 28–31). Thus, it has been largely held that dysfunctional mitochondrial FAO promotes AKI and that augmenting mitochondrial FAO will protect against injury and accelerate recovery (32–34). Agents that indirectly stimulate mitochondrial function and FAO, such as PGC1α overexpression (35), the AMPK agonist AICAR, PPARα agonist drugs, and a compound called C75 that is believed to activate a critical FAO enzyme known as carnitine palmitoyltransferase-I (CPT1), have all been associated with protection against AKI (36–38). At the same time, etomoxir, an irreversible inhibitor of CPT1 that strongly inhibits mitochondrial FAO, also protects against AKI (39). Thus, the role of mitochondrial FAO in protection versus exacerbation of renal injury in AKI is unclear.

These apparently discrepant data regarding mitochondrial FAO during AKI might be reconciled by considering peroxisomal FAO. RTECs are also rich in peroxisomes, which possess a biochemically parallel FAO pathway to that of the mitochondria that does not directly produce ATP. While PPARα agonists increase expression of mitochondrial FAO genes, their effects on the peroxisomal FAO gene expression are much greater, at least in rodents (40). Agents that have been shown to protect against AKI, such as AICAR and C75, all activate PPARα and will thus increase peroxisomal biogenesis and FAO (39, 41–45). PGC1α has also been shown to increase peroxisomal abundance and activity (46). There appears to be considerable crosstalk and cooperation between the peroxisomal and mitochondrial FAO pathways. Some substrates are partially chain-shortened in peroxisomes and then passed on to mitochondria (47). Furthermore, inhibiting flux through mitochondrial FAO causes a compensatory increase in peroxisomal FAO and vice versa (48, 49).

Mitochondrial ROS is well known to be a driver of numerous disease states in the kidney, and FAO is the largest contributor to this ROS. In one study of diabetic kidney, the pathological increase in ROS was ascribed completely to FAO (3). Importantly, the source of this ROS was found to be upstream of the electron transport chain. The leaked electrons were coming either directly from the acyl-CoA dehydrogenases, which catalyze the first step in β-oxidation, or from their redox partners ETF and ETF dehydrogenase. Others have shown that ETF and ETF dehydrogenase can leak electrons (50, 51), and we have shown that, among the acyl-CoA dehydrogenases, it is LCAD that is most susceptible to electron leakage to oxygen. LCAD is highly specific for mitochondrial FAO, but not for peroxisomal FAO, in mouse and human kidneys (52). LCAD has a wider substrate-binding pocket than other acyl-CoA dehydrogenase family members, which allows oxygenated solvent partial access to the electrons. The result is electrons jumping to oxygen and direct formation of H_2_O_2_. While LCAD downregulation is observed in many cancers to reduce H_2_O_2_ levels (11, 12), reexpression of LCAD in liver cancer cell lines by stable transfection increases ROS (12).

**Figure 9:**
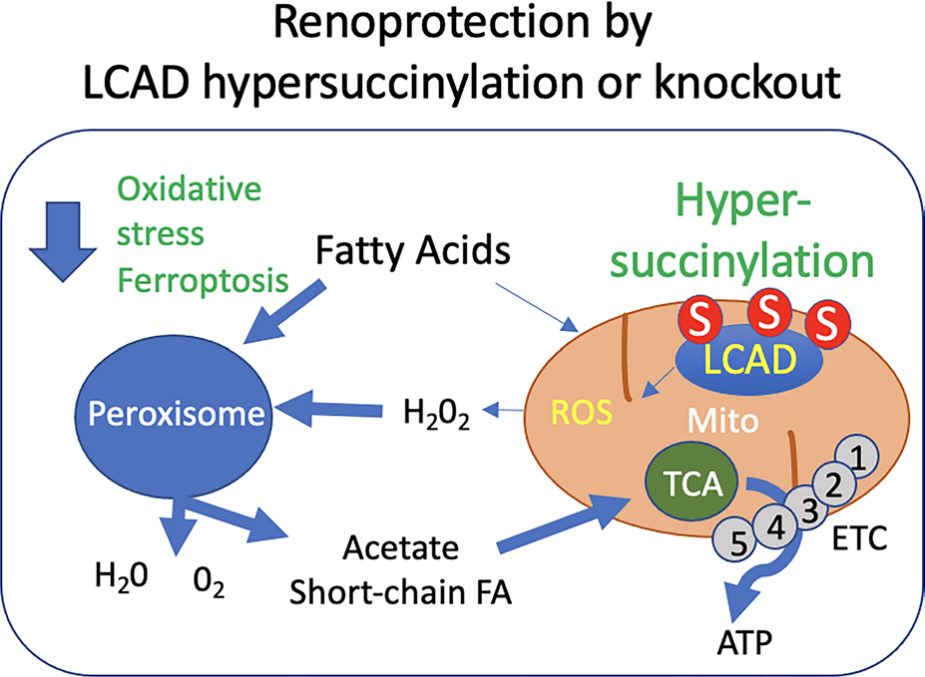
Proposed model by which loss of or hypersuccinylation of LCAD mediates renoprotection against AKI. Enhanced peroxisomal activity due to loss of LCAD reduces H_2_O_2_ production and contributes to renoprotection against AKI. Ferroptotic cell death (ROS-associated form of cell death) is attenuated in *LCAD^–/–^* kidneys. S, hypersuccinylation.

Here, we showed that partial suppression of mitochondrial FAO by ablation of LCAD was sufficient to reduce the severity of tissue injury induced by 2 forms of AKI (Figure 9). Renoprotection by *LCAD^–/–^* against the nephrotoxicity of cisplatin was independent of sex differences. A potentially greater capacity for FAO in female kidneys than in males has been suggested for their protective advantage against AKI (53, 54). The sirtuin family proteins play a crucial role in regulating FAO. Indeed, female-specific renoprotection against IRI was associated with higher levels of sirtuin 3 in female kidneys (55, 56). Male kidneys decrease sirtuin 6 expression after AKI, which contributes to exacerbated AKI compared with female kidneys (57). Interestingly, LCAD is hyperacetylated in the *Sirt3^–/–^* liver during the fasting state (58). Furthermore, scRNA-Seq analysis revealed that LCAD is highly expressed in the S3 area of proximal tubules in healthy mouse kidneys, especially in males (Supplemental Figure 2). This suggests that LCAD may play a role in the sex difference in some conditions.

The mechanism of protection may involve a reduction in mitochondrial ROS coincident with increased flux of fatty acids through the peroxisomal FAO pathway. The level of acylcarnitine (the main transporter of long-chain fatty acids to the mitochondria) was not altered in *LCAD^–/–^* kidneys (59). Our data for mitochondrial respiration showed protected mitochondrial function in *LCAD^–/–^* kidneys after AKI, while the level of renal ROS is decreased. The peroxisomal FAO pathway also produces H_2_O_2_, but peroxisomal catalase reverts the H_2_O_2_ back to O_2_ and water. The action of catalase, which in essence regenerates oxygen, allows FAO to proceed with a reduced oxygen cost, which could be particularly beneficial during ischemic AKI. Future studies are needed to more accurately quantify the degree of peroxisomal FAO induction in proximal tubules upon ablation of the mitochondrial pathway.

Translationally, LCAD may be an attractive target for AKI intervention. In rodents, LCAD is broadly expressed and plays a critical role in tissues where mitochondrial FAO is indispensable for energy production, such as heart and muscle. In humans, however, LCAD expression is restricted to kidney, liver, lung, and pancreas (10), such that inhibition of LCAD in humans would not compromise heart and muscle as it would in rodents. Studies are underway to determine the optimal means by which to exploit this mechanism for the prevention and treatment of AKI.

One of the limitations of the present study is largely dependent on the mouse model of LCAD global KO. We have previously shown that dietary supplementation with octanedioic acid (8-carbon dicarboxylic acids) enhances peroxisomal FAO in a highly kidney-specific manner to protect against AKI (16). A cell type–specific loss-of-function study will help to better understand the role of LCAD in AKI.

## Methods

### Sex as a biological variable.

Male mice were used for both cisplatin and renal IRI AKI models throughout the manuscript. Both male and female mice were studied in the cisplatin AKI model to confirm similar injury phenotypes.

### LCAD protein succinylation and H_2_O_2_ assay.

Recombinant mouse LCAD or its mutant K322R were succinylated with 0.1 mM succinyl-CoA in MSH buffer (210 mM mannitol, 70 mM sucrose, 5 mM Hepes [pH 8.0]). For control proteins, the same volume of succinyl-CoA solvent 20 mM sodium acetate (pH 6) was added in MSH buffer. After 30 minutes incubation at 37°C, the buffer was changed to 50 mM NaPO4 (pH 7.4) with 10% glycerol, and the proteins were concentrated using Amicon Centrifugal filters.

For H_2_O_2_ assay, Amplex UltraRed reagent (Invitrogen, A36006), 96-well black microplate and a FLUDstar Omega plate reader (BMG LABTECH), and assay buffer (20 mM NaPO4 [pH7.4]) were used for the assay. Briefly, 50 mL of work solution (100 μM Amplex UltraRed and 2 unit of Horseradish peroxidase in assay buffer), 2 mg of mouse LCAD or its mutant K322R, and assay buffer was added to total 95 mL for each reaction. The assay was started when 5 mL of 1 mM palmityl-CoA (final 50 mM) was added to the reaction solution in the 96-well plate on the plate reader. The measurements were taken using fluorescence emission/excitation settings of 520/580 nm and lasted for 30 minutes.

### scRNA-Seq data for human and mouse kidney.

scRNA-Seq data for *Acadl* (LCAD) expression were obtained from the publicly available Kidney Precision Medicine Project (KPMP) Kidney Tissue Atlas (https://atlas.kpmp.org/explorer/dataviz) and from KidneyCellExplorer (https://cello.shinyapps.io/kidneycellexplorer/) website (60) for human kidneys as well as from Katalin Susztak’s Kidney Biobank multi-omics datasets (https://susztaklab.com/) for mouse kidneys (61).

### Succinylome analysis of Sirt5^–/–^ and WT kidney tissues.

We have previously reported the quantitative analysis of protein succinylation in WT uninjured versus injured kidneys (*n* = 3) (16) or in *Sirt5^–/–^* versus WT (*n* = 3) mouse kidney tissues collected after renal IRI (17). Briefly, kidney tissues were homogenized, trypsinized, desalted, and further enriched for succinylated peptides using the PTMScan Succinyl-Lysine Motif Kit (Cell Signaling Technologies) for succinyl-lysine PTM analysis. Samples were analyzed by data-independent acquisition on a TripleTOF 6600 system. The peptide AFG^322^KsuccTVAHIQTVQHK with a succinyl group on the lysine residue K322 of the mitochondrial LCAD was analyzed in Skyline (62). The extracted ion chromatograms of the peptide AFG^322^KsuccTVAHIQTVQHK (precursor ion at *m/z* 441.99, *z* = 4+) were manually assessed, and we confirmed accurate peak integration boundaries and removed potentially interfered transitions. Quantification and statistics were performed with Skyline. Briefly, peptide quantification was obtained by summing the transition area, and a 2-tailed *t* test was applied to compare AFG^322^KsuccTVAHIQTVQHK peptide abundance level in *Sirt5^–/–^* versus WT replicates.

### Animals.

*LCAD^–/–^* mice were obtained from the Mutant Mouse Regional Resource Center. The *LCAD^–/–^* mice on a mixed background of C57BL/6 and 129 were crossed with a C57BL/6 to generate heterozygous *LCAD^+/–^* mutants. Homozygous *LCAD^–/–^* mutants or WT controls (*LCAD^+/+^*) were derived from the common breeding pairs of *LCAD^+/–^* heterozygous mutants and used throughout the current study. All animals utilized for experiments were between 10 and 14 weeks of age. All experiments were conducted in males except where noted.

### Renal IRI induced AKI model.

Renal IRI was induced for the male mice by a unilateral renal IRI with a delayed contralateral nephrectomy as previously described (16, 17, 63). Briefly, mice were anesthetized with 2% isoflurane. Core body temperature of the mice was monitored with a rectal thermometer probe and was maintained at 36.8°C–37.2°C throughout the procedures with a water-heating circulation pump system (EZ-7150; BrainTree Scientific) and an infrared heat lamp (Shat-R-Shield). Extended-release buprenorphine 1.3 mg/mL (Ethiqa XR, Fidelis MIF 900-014) was administered for analgesia (3.25 mg/kg body weight [b.w.], s.c.). With aseptic techniques, a dorsal incision was made to expose the left kidney, and renal ischemia for 18 minutes was induced by clamping of the left kidney pedicle with a nontraumatic micro serrefines (18055–04; Fine Science Tools). Renal reperfusion was visually verified afterward. Contralateral nephrectomy of the right kidney was performed at day 6 after renal IRI. Serum and the injured left kidney were collected at day 7. Serum was analyzed by the Kansas State Veterinary Diagnostic Laboratory for Renal Profile, which include analysis of albumin, BUN, creatinine, and phosphorus.

### Cisplatin nephrotoxic AKI model.

To induce cisplatin AKI, male and female mice were given a single dose of cisplatin (20 mg/kg b.w., i.p. for females and 24 mg/kg b.w., i.p. for males; Fresenius Kabi), or vehicle control of normal saline as described previously (16, 17). Serum and kidneys were collected on day 3 post-cisplatin.

### Cultured RTECs.

Primary renal tubules (both LCAD KO and WT littermates) were plated on coverslips in a 12-well tissue culture plate in DMEM:F12 media at 37°C and 5% CO_2_. Once the cells were attached to the coverslips, the media was removed, the cells were rinsed in 1× PBS before being incubated overnight in Glucose-free media (115 mM NaCl, 1.0 mM NaH_2_PO_4_, 26.2 mM NaHCO_3_, 5.4 mM KCl, 1.8 mM CaCl_2_, and 0.8 mM MgSO_4_) and 1% O_2_. The following day, the cells were placed back into normal media and Oxygen concentrations for 24 hours. The MitoSOX reagent was prepared per manufacturer’s recommendation (diluted in DMSO to a stock concentration of 5 mM; Thermo Fisher Scientific, M36008). The reagent was added to cells and incubated for 30 minutes at 37°C and then washed out into HBSS without Phenol red. The live cells in the tissue culture dish were imaged on the Leica DMi8, and average gray value intensity of the 594 channel was compared between LCAD^–/–^ or LCAD^+/+^ cells to assay SuperOxide activity and oxidative stress.

For immunofluorescence, 24 hours after combined glucose and oxygen deprivation, the cells were fixed on to the coverslips in 4% paraformaldehyde (Electron Microscopy Sciences, 15710) for 10 minutes then washed in PBS. The fixed cells were permeabilized with 0.3% Triton-PBS for 5 minutes, washed in PBS, then blocked in 10% donkey serum (MilliporeSigma, D9663), 0.05% Tween-20 PBS (MilliporeSigma, 11332465001) for 1 hour at room temperature. Primary antibodies (diluted in block) were polyclonal Trx (Invitrogen, PA590044) and mouse 4-HNE (Invitrogen, MA527570). After overnight incubation in primary antibodies (1:100), coverslips were incubated with anti–mouse Alexa Fluor 488–conjugated and anti–rabbit Alexa Fluor 594–conjugated secondary antibodies (1:200).

Immunofluorescence was imaged on a Leica DMi8 inverted wide-field scope at 20× magnification. Image quantification was performed on raw images using ImageJ/FIJI software. Briefly, a mask was created to isolate the region of interest around the tubules, removing any background area. The average gray-value intensity for each region of interest was recorded in PRISM software and graphed to compare oxidative stress in WT and LCAD-KO tubules.

### Western blotting.

Kidney tissues were lysed in RIPA buffer (Thermo Fisher Scientific, 89901), and the homogenates were plated in triplicate to measure protein concentration using a Bradford assay kit (Bio-Rad). Western blotting analysis was performed as previously described (17). The following commercially available antibodies were used in this study: anti-GPX4 (Abcam, ab125066), anti-PEX5 (Thermo Fisher Scientific, PA5-58716), anti-PMP70 (Abcam, ab85550; 1:500), and SDHA (Cell Signaling Technology, 11998; 1:1,000) and loading controls GAPDH (Sigma-Aldrich, G8795; 1:500) and HSP60 (Cell Signaling Technology, 12165; 1:1,000). The rat LCAD antibody (1:5,000) was provided by Jerry Vockley at UPMC CHP (1:1,000) and was previously characterized by us (10). Immunoblots were subjected to densitometric analysis using ImageJ software (NIH).

### Real-time PCR.

Real-time PCR analysis was performed as previously described to determine mRNA abundance (17). cDNA was reverse transcribed from 500 ng of total RNA with SuperScript First-Strand Synthesis System II (Thermo Fisher Scientific). Real-time PCR analysis was performed with gene specific primer oligos, SsoAdvanced SYBR Green Super-mix (Bio-Rad, 1725271) and CFX96 Touch Real-Time PCR Detection System with C1000 Thermal Cycler (Bio-Rad). Cycling conditions were 95°C for 10 minutes, followed by 40 cycles of 95°C for 15 seconds and 60°C for 1 minute. Data were normalized to *Rn18S* as an endogenous control and analyzed using the 2^–ΔΔCT^ method. Each primer sequence is shown in Supplemental Table 2.

### Tissue section analysis.

Kidneys were fixed in 4% paraformaldehyde and embedded in OCT or paraffin. These tissues were sectioned at 10 μm or 4 μm, respectively. The kidney sections were stained with H&E. H&E-stained slides were scored semiquantitatively (from 0 to 4) for tubular damage in a blinded fashion with respect to tubular dilation, proteinaceous cast formation, and loss of brush border. Renal cortex and outer medulla regions were scored separately.

Immunostaining was performed as previously described (17), with OCT- or paraffin-embedded tissues and with following primary antibodies: anti-KIM1 (R&D, MAB1817), anti-LCAD, anti-NGAL (R&D, AF1857), anti-PEX5 (Cell Signaling Technology, 830205; 1:50), anti-PMP70 (Abcam, ab85550; 1:50), anti-SDHA (Cell Signaling Technology, 11998; 1:200), or LTL (Vector Laboratories, FL-1321; 1:100). Antibodies were used at 1:50–1:200, followed by conjugation with Alexa Fluor antibodies at 1:200.

TUNEL staining was performed by using the In Situ Cell Death Detection Kit (Roche, 12156792910) to evaluate cell death in paraffin-embedded kidney tissue according to manufacturer instructions. TUNEL^+^ cells are quantified by ImageJ as previously described (64). Most of the images were obtained with a Leica DM2500 optical microscope. LCAD immunofluorescence images were obtained with a Leica TCS SP8 is an inverted laser scanning confocal microscope.

### ETF fluorescence reduction assay.

ETF fluorescence reduction assay analysis was performed to examine LCAD activity on FLUOstar Omega fluorescence spectrophotometer plate reader (BMG Labtech) as previously described (65). Briefly, each 200 μL of assay reaction contained 50 μg kidney lysate and 25 μM palmitoyl-CoA. The reaction buffer contained 50 mM Tris-HCl (pH 8.0), 0.5% glucose, 2 μM recombinant pig ETF, and 1.43 μL of glucose oxidase/catalase mixture. The remaining activity of the KO mice should be from VLCAD, for which palmitoyl-CoA is also the favorable substrate. 

### Oroboros high-resolution respirometry.

Freshly prepared kidney homogenates were analyzed with an Oroboros Oxygraph-2K using our previously published method (17). Complex I respiration was defined as malate/ pyruvate/glutamate-driven oxygen consumption in the presence of ADP, whereas Complex II respiration was defined as succinate-driven oxygen consumption.

### mtDNA analysis.

Total DNA was extracted using the DNeasy Blood & Tissue kit (Qiagen, 69504). mtDNA was quantified through a TaqMan-based assay as previously described (66–68). Two sets of PCR primers, targeting the mitochondrial NADH dehydrogenase 1 (mt-ND1) gene and the histone deacetylase 1 (Hdac1) gene, were utilized to amplify the mtDNA and the nuclear DNA (nDNA), respectively (69). PCR were performed on a CFX96 Touch Real-Time PCR Detection System (Bio-Rad) under the following conditions: 95°C for 180 seconds, 45 cycles at 95°C for 15 seconds, and 60°C for 60 seconds. mtDNA copy number relative to the nDNA was calculated using the 2^–ΔΔCT^ method.

### F_2_-isoPs and isofurans analysis.

F_2_-IsoPs and isofurans were measured in the Vanderbilt University Eicosanoid Core Laboratory after lipid extraction from snap-frozen kidney samples using gas chromatography–mass spectrometry, as previously described (25).

### Statistics.

Data are presented as mean ± SD. Prism 9.0.0 software (GraphPad) was used for statistical analysis. To determine whether sample data has been drawn from a normally distributed population, D’Agostino-Pearson omnibus test or Shapiro-Wilk test was performed. For parametric data, 1-way ANOVA with post hoc Tukey comparison was used for multiple-group comparison and 2-tailed Student’s *t* test was used to compare 2 different groups. For nonparametric data, Mann-Whitney *U* test was used. A *P* value less than 0.05 was considered significant.

### Study approval.

All animal protocols were approved by the University of Pittsburgh IACUC (protocol no. 22112009) at the Office of Research Protection/Animal Research Protection in Pittsburgh, Pennsylvania, USA. All experiments were conducted in accordance with the guidelines and regulations of the Animal Welfare Act (AWA) and the PHS Policy on Humane Care and Use of Laboratory Animals.

### Data availability.

The Supporting Data Values file presenting each statistical analysis is available in the supplemental materials. The data are publicly available at the Center for Computational Mass Spectrometry, (the MassIVE repository at UCSD) and can be downloaded at https://massive.ucsd.edu/ProteoSAFe/dataset.jsp?task=6dfcd4ae92a7471a85fa0bf0add8c9bd (MassIVE ID: MSV000091214; ProteomeXchange ID: PXD039904) and Mass Spectrometry Interactive Virtual Environment (https://massive.ucsd.edu/ProteoSAFe/dataset.jsp?task=e33b6976d0f14c268e8a48e3a3ae84d8; with MassIVE ID: MSV000083439; ProteomeXchange ID: PXD012696).

## Author contributions

TC and AO designed and performed experiments, provided intellectual input, and wrote the manuscript. YZ, JB, SSB, KP, BBZ, AK, and AR performed experiments and provided intellectual input. BS, EG, and SSL designed and performed experiments, edited the manuscript, provided intellectual input, and oversaw the project. All authors approved the final version of this manuscript.

## Supplementary Material

Supplemental data

Unedited blot and gel images

Supporting data values

## Version 1 02/11/2025

In-Press Preview

## Version 2 03/24/2025

Electronic publication
