# Supplementary material for: Loss of long-chain acyl-CoA dehydrogenase protects against acute kidney injury
Source: JCI Insight. 2025 Feb 11;10(6):e186073. doi: 10.1172/jci.insight.186073 (PMC11949023; doi:10.1172/jci.insight.186073)
Supplement: Supplemental data [file jciinsight-10-186073-s325.pdf]

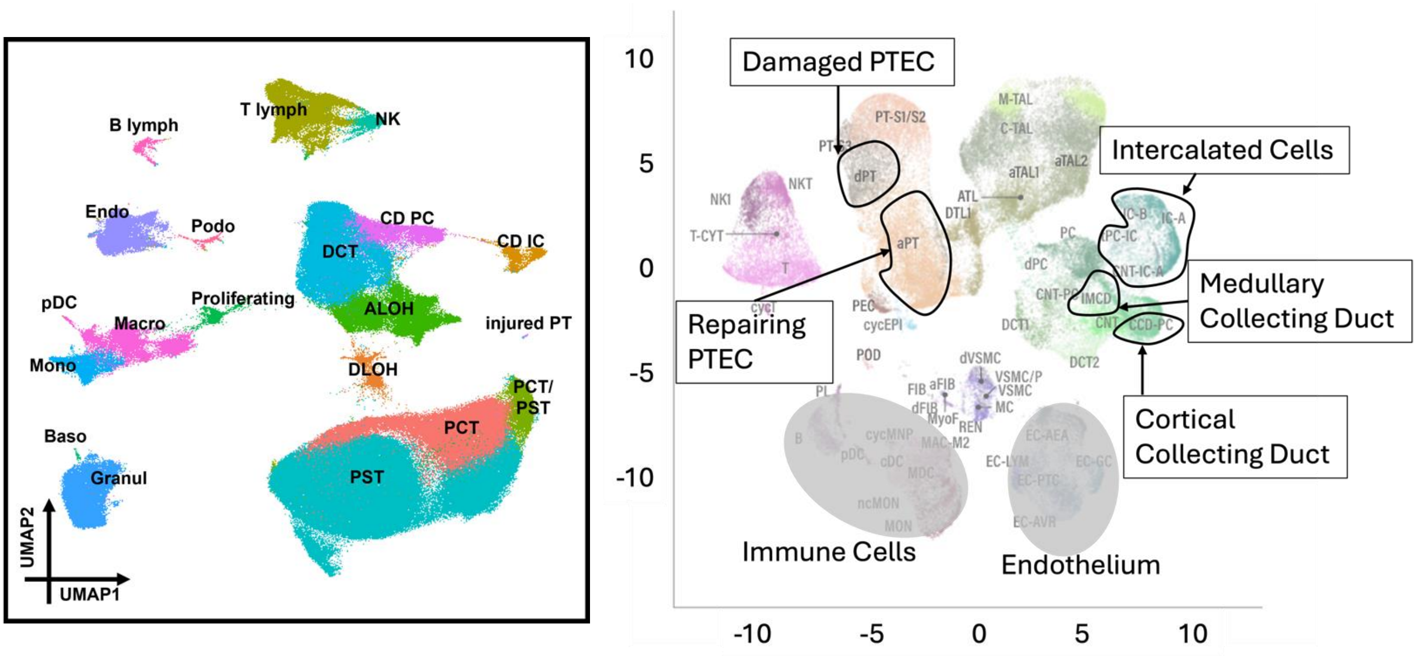

Supplemental Figure 1. Spatial localization of *ALCAD* (LCAD) in renal cell clusters from healthy and AKI mouse and human kidneys. (A) UMAP of 280,521 mouse kidney single cells. Nineteen cell types were identified: ALOH, ascending loop of Henle; B lymph, B lymphocyte; Baso, basophile; CD IC, collecting duct intercalated cell; CD PC, collecting duct principal cell; DCT, distal convoluted tubule; DLOH, descending loop of Henle; Endo, endothelial cell; Granul, granulocyte; injured PT, injured proximal tubule; Macro, macrophage; Mono, monocyte; NK, natural killer cell; PCT, proximal convoluted tubule; pDC, plasmacytoid dendritic cell; Podo, podocyte; Proliferating, proliferating cell; PST, proximal straight tubule; T lymph, T lymphocyte. (B) UMAP of data were derived from the Kidney Precision Medicine Project (KPMP) Kidney Tissue Atlas. Healthy; n = 28. AKI; n=14, CKD; n = 37. The results here are in whole or part based upon data generated by KPMP. November 1 2024. https://www.kpmp.org.

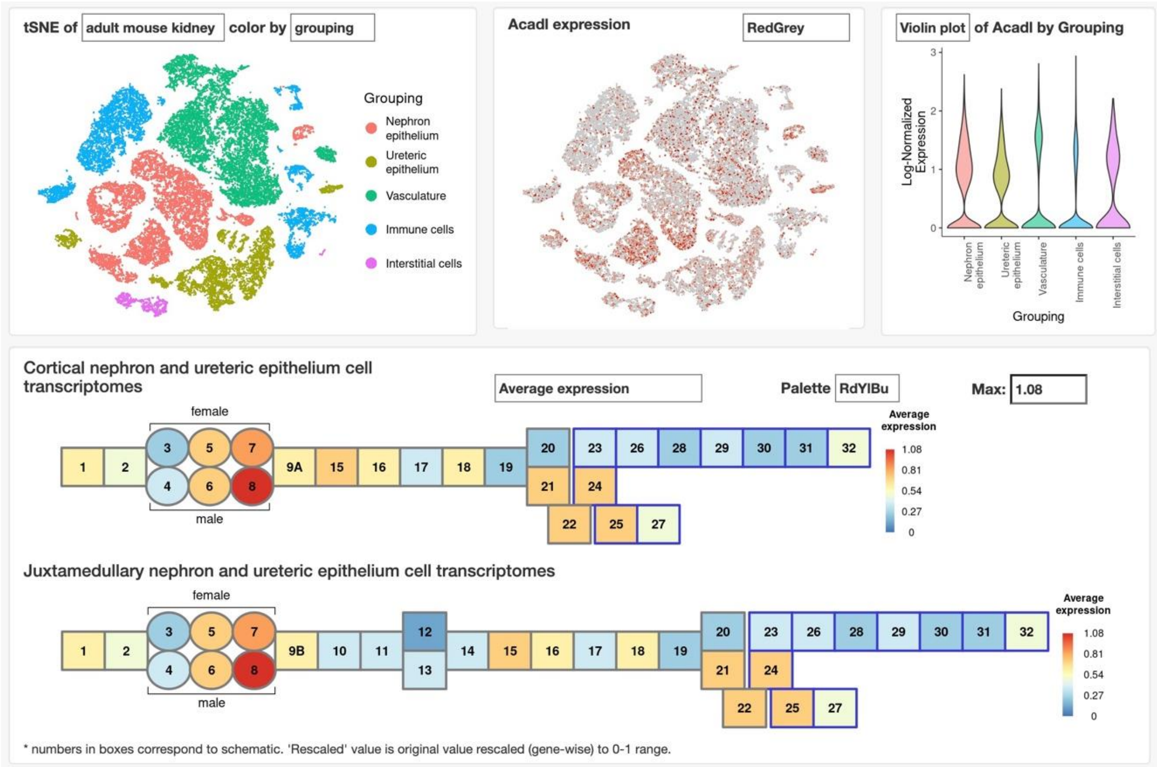

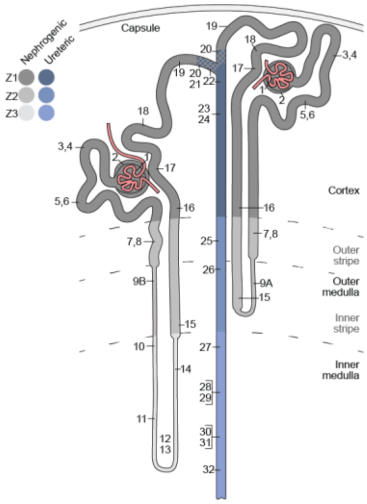

Supplementary Figure 2. Differential expression of *Acadl* (LCAD) in renal cell clusters from healthy male and female mouse kidneys. Data from KidneyCellExplorer (https://cello.shinyapps.io/kidneycellexplorer/ Ransick et al., 2019).

1. podocytes (visceral epithelium) 2. parietal epithelium 3. segment 1 of proximal tubule – female 4. segment 1 of proximal tubule – male 5. segment 2 of proximal tubule – female 6. segment 2 of proximal tubule – male 7. segment 3 of proximal tubule – female 8. segment 3 of proximal tubule – male 9A. LOH thin descending limb of inner stripe of outer medulla of cortical nephron 9B. LOH thin descending limb of inner stripe of outer medulla of juxtamedullary nephron 10. upper LOH thin descending limb of inner medulla of juxtamedullary nephron 11. lower LOH thin descending limb of inner medulla of juxtamedullary nephron12. lower LOH thin limb of inner medulla of juxtamedullary nephron 13. lower LOH thin limb of inner medulla of juxtamedullary nephron 14. upper LOH thin ascending limb of inner medulla of juxtamedullary nephron 15. distal straight tubule of inner stripe of outer medulla (syn: thick ascending limb of LOH) 16. distal straight tubule of outer stripe of outer medulla and cortex (syn: thick ascending limb of LOH) 17. macula densa 18. distal convoluted tubule 19. nephron connecting tubule 20. principal-like cell of nephron connecting tubule 21. intercalated type non-A non-B cell of nephron connecting tubule 22. intercalated type A cell of nephron connecting tubule and cortical collecting duct 23. principal-like cell of cortical collecting duct 24. intercalated type B cell of cortical collecting duct 25. intercalated type A cell of outer medullary collecting duct 26. principal cell of outer medullary collecting duct 27. intercalated type A cell of inner medullary collecting duct 28. principal cell of inner medullary collecting duct type 1 29. principal cell of inner medullary collecting duct type 2 30. principal-like cell of deep inner medullary collecting duct type 1 31. cell of deep inner medullary collecting duct type 2 32. deep medullary epithelium of pelvis

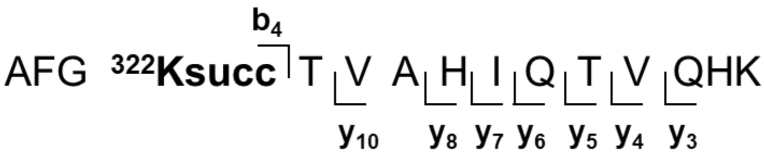

A. XIC of *Sirt5*$^{-/-}$, post-AKI

B. XIC of WT, post-AKI

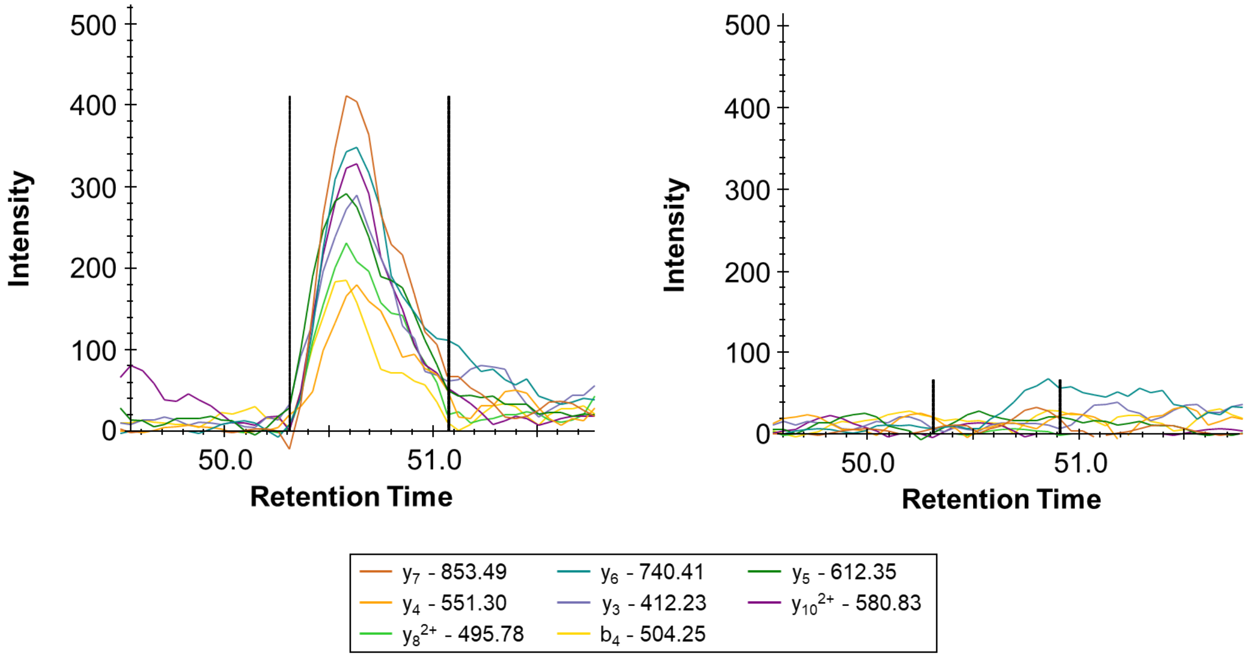

C. Quantification of ^{322}Ksucc

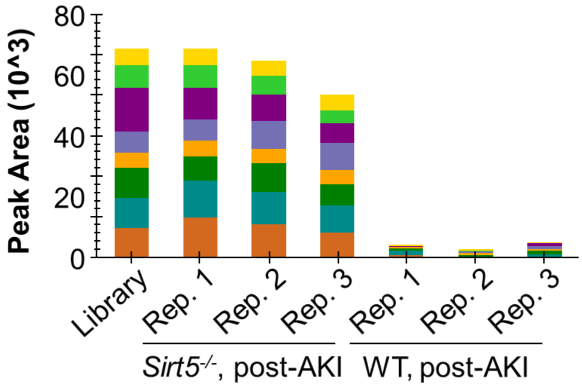

D. Statistics: *Sirt5*$^{-/-}$ vs WT for ^{322}Ksucc

**Fold-change (*Sirt5*$^{-/-}$ vs WT)**	**Log_2 (*Sirt5*$^{-/-}$ vs WT)**	**p-value**
16.5	4.04	0.0003

n = 3 for each condition

Supplementary Figure 3. Mass spectrometry revealed hypersuccinylation of lysine K322 of LCAD in *Sirt5*$^{-/-}$ kidneys post-AKI. Extracted ion chromatograms (XICs) of the peptide AFG**^{322}Ksucc**TVAHIQTVQHK (precursor ion at m/z 441.99, z = 4+) of LCAD in kidney tissues from a (A) a *Sirt5*$^{-/-}$ biological replicate and a (B) WT biological replicate, post-AKI. (C) Quantification of the succinylated peptide in the three *Sirt5*$^{-/-}$ replicates and the three WT replicates, showing peak areas as determined in Skyline. (D) Statistical analysis confirmed the increased succinylation level of K322 of LCAD in *Sirt5*$^{-/-}$ vs WT kidneys, post-AKI.

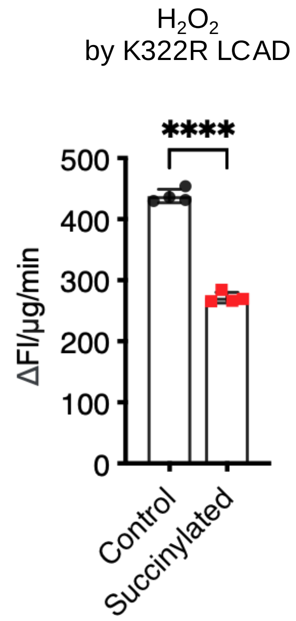

Supplementary Figure 4. Succinylation reduces oxidase activity of K322R mutant LCAD protein. n=4, ****p<0.0001. *t*-test

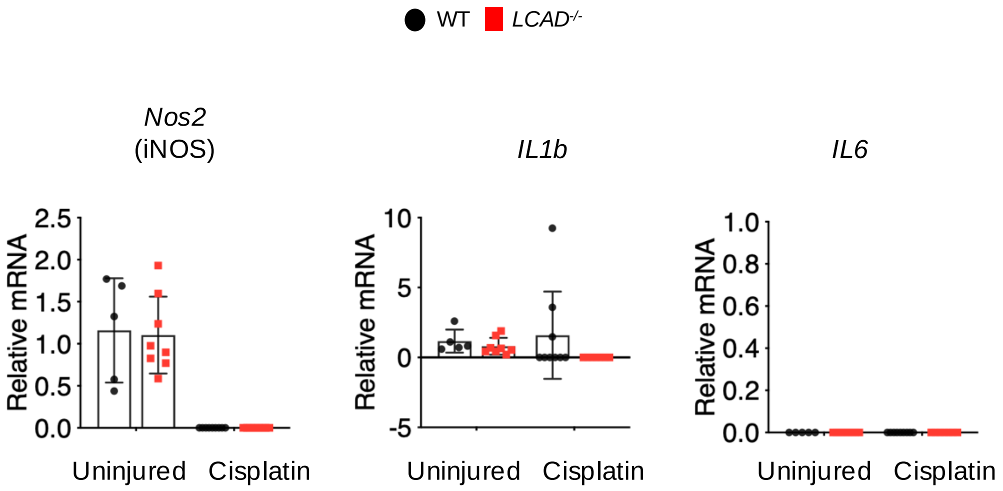

Supplementary Figure 5. mRNA levels of inflammatory macrophage markers in *LCAD*$^{-/-}$ after cisplatin-AKI.
n=5-9. One-way ANOVA *post hoc* Tukey multiple comparison

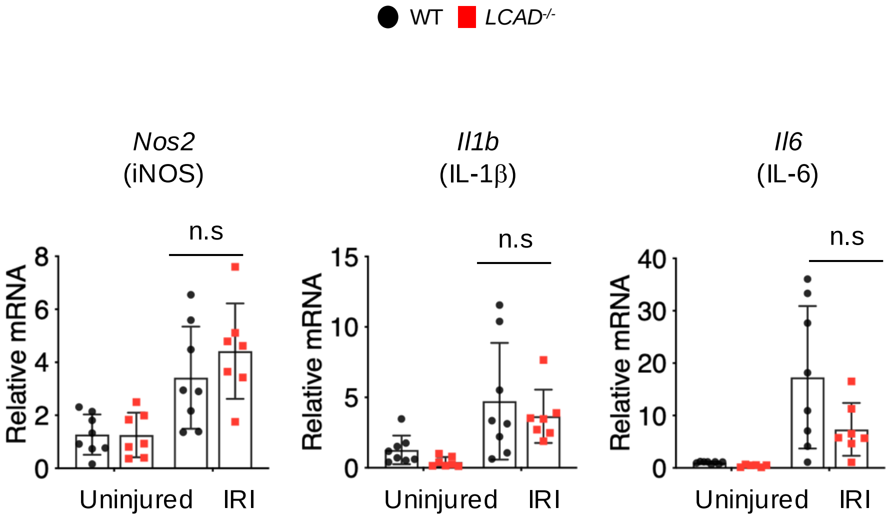

Supplementary Figure 6. mRNA levels of inflammatory macrophage markers in *LCAD*$^{-/-}$ after renal IRI. n=7-8. One-way ANOVA *post hoc* Tukey multiple comparison

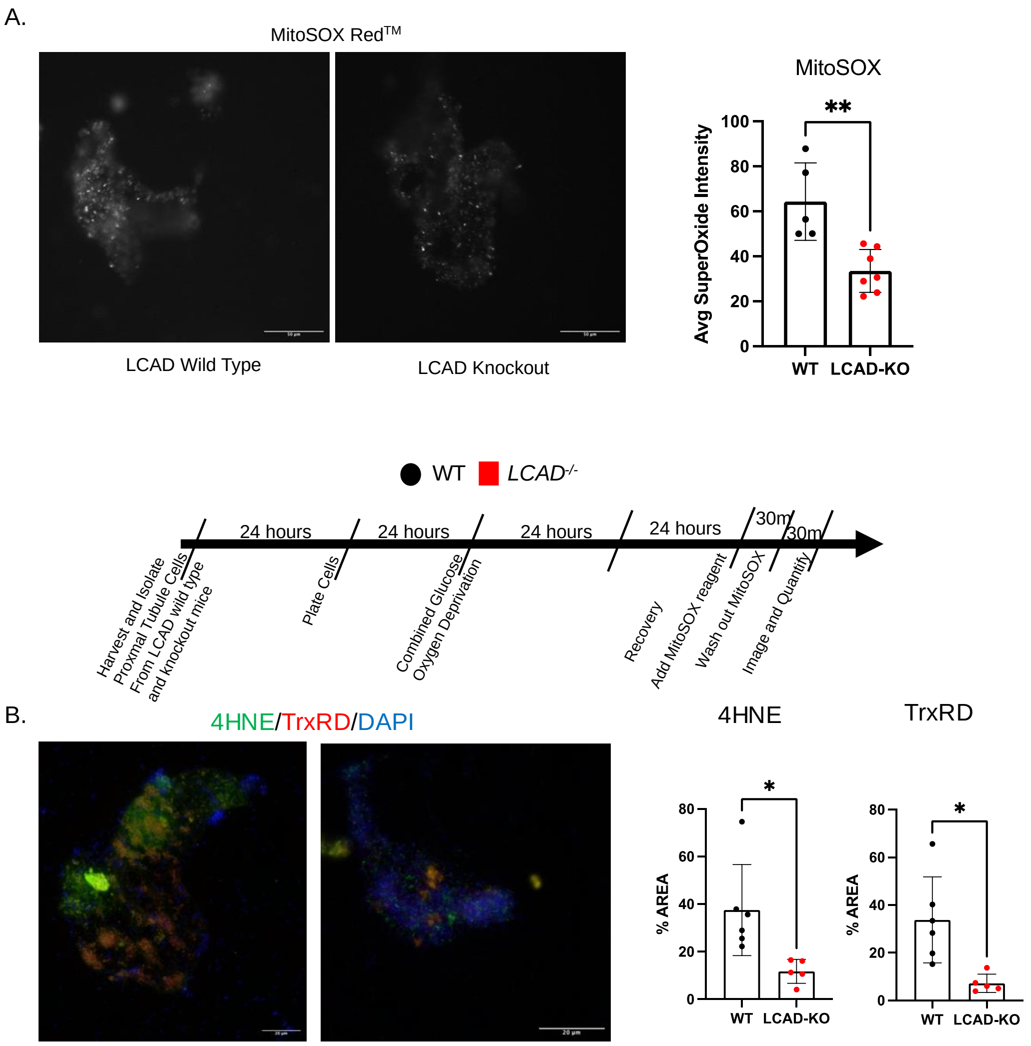

Supplementary Figure 7. Oxidative stress was decreased in *$LCAD^{-/-}$* proximal tubules after CGOD. Proximal tubules from LCAD-/- and controls grown in the presence of CGOD. A. MitoSOX fluorescence intensity staining showing a decrease in MitoSOX activity in the LCAD-/- tubules compared to controls. B. Immunofluorescence staining for oxidative stress markers 4HNE and TrxRD depicting the decreased intensity and less oxidative stress in the LCAD-/- compared to controls. n=5-6. Student T-Tests used for analysis.

Cell Type		#cells	mean expression	%cells expressing	Fold change	P Value
**Healthy**						
PTEC (adaptive/repairing)	**aPT**	2499	0.546	21.5	1.96	2.88E-45
PTEC degenerative	**dPT**	1274	0.297	13.9	0.9	3.93E-08
Distal Tubule Type 1	**DCT1**					
Distal Tubule Type 2	**DCT2**					
Inner Medullary Collecting Duct	**IMCD**	742	0.476	32.5	1.6	1.02E-62
Cortical Collecting Duct	**CCD-PC**	1064	0.404	22.3	1.36	6.59E-34
Glomerular Capillary Endothelial	**EC-GC**					
Vascular smooth muscle cell	**VSMC**					
Intercalated Cells Type A	**IC-A**	1980	0.409	24.8	1.43	7.22E-54
Connecting Tubule Intercalated Cell Type A	**CNT-IC-A**	712	0.323	27.9	1.01	1.30E-43
Descending Thin Limb	**DTL1**	253	0.791	23.7	2.32	8.49E-16
Connecting Tubule Cell	**CNT**	1632	0.203	12.2	0.328	0.0003
Parietal Epithelial Cell	**PEC**	242	0.298	14	0.892	0.0005
Principal Cell	**PC**	960	0.273	11.4	0.77	0.000667
**AKI**						
	**aPT**	8265	0.523	18.6	1.59	1.12E-38
	**dPT**					
	**DCT1**					
	**DCT2**					
	**IMCD**					
	**CCD-PC**	999	0.664	22.8	1.59	2.78E-32
	**EC-GC**					
	**VSMC**					
	**IC-A**	858	0.522	22.7	1.22	1.55E-31
	**CNT-IC-A**	281	0.497	24.2	1.13	6.29E-17
	**DTL1**	1555	0.618	14.5	1.51	3.27E-10
	**CNT**					
	**PEC**					
	**PC**	1462	0.325	13.5	0.516	9.31E-08

Supplemental Table 1. Differential expression of *Acadl* (LCAD) in renal cell clusters from healthy and AKI human kidneys. Data were derived from the Kidney Precision Medicine Project (KPMP) Kidney Tissue Atlas. Healthy; n = 28. AKI; n=14, CKD; n = 37. Note: PTEC (adaptive/repairing) represents successful or failed repair. The results here are in whole or part based upon data generated by KPMP. November 1 2024. https://www.kpmp.org.

Gene	Fw	Rv
Abcd3 (PMP70*)*	CTGACCAGGTGCTGAAGGAG	CCTCCACATCCACACTGACC
Acox1	TAACTTCCTCACTCGAAGCCA	AGTTCCATGACCCATCTCTGTC
Alox5	ACTACATCTACCTCAGCCTCATT	GGTGACATCGTAGGAGTCCAC
Ehhadh	ATGGCTGAGTATCTGAGGCTG	GGTCCAAACTAGCTTTCTGGAG
Gpx4	GCCTGGATAAGTACAGGGGTT	CATGCAGATCGACTAGCTGAG
IL1b	ACAAGGAGAACCAAGCAACG	ACTCTGCAGACTCAAACTCCAC
IL6	ACAAAGCCAGAGTCCTTCAGAG	TTGGATGGTCTTGGTCCTTAGC
Lcn2 (NGAL*)*	GCAGGTGGTACGTTGTGGG	CTCTTGTAGCTCATAGATGGTGC
Nos2	GCAGGTGGTACGTTGTGGG	ACCCAAACACCAAGCTCATG
Ptges2	CCTCGACTTCCACTCCCTG	TGAGGGCACTAATGATGACAGAG
Rn18S	AGAAACGGCTACCACATCCA	TACAGGGCCTCGAAAGAGTC

Supplementary Table 2. Primer sequences for qPCR
