# Supplementary figures and images for: Loss of long-chain acyl-CoA dehydrogenase protects against acute kidney injury
Source: JCI Insight. 2025 Feb 11;10(6):e186073. doi: 10.1172/jci.insight.186073 (PMC11949023; doi:10.1172/jci.insight.186073)

# Uncropped Western blot images

## Figure 1A (LCAD)

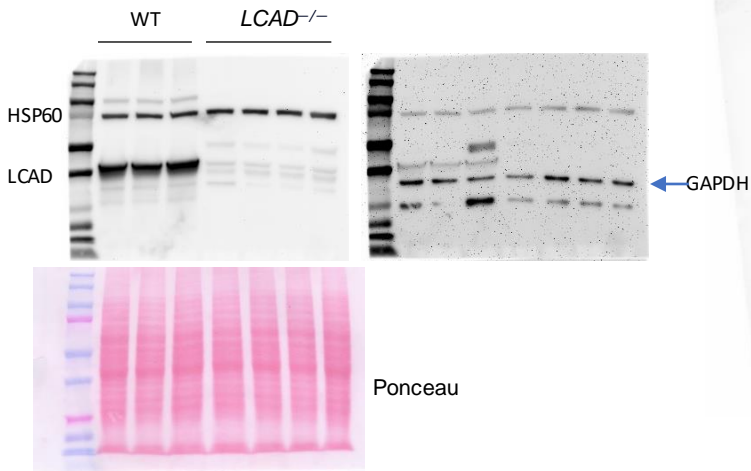

## Figure 7C (SDHA)

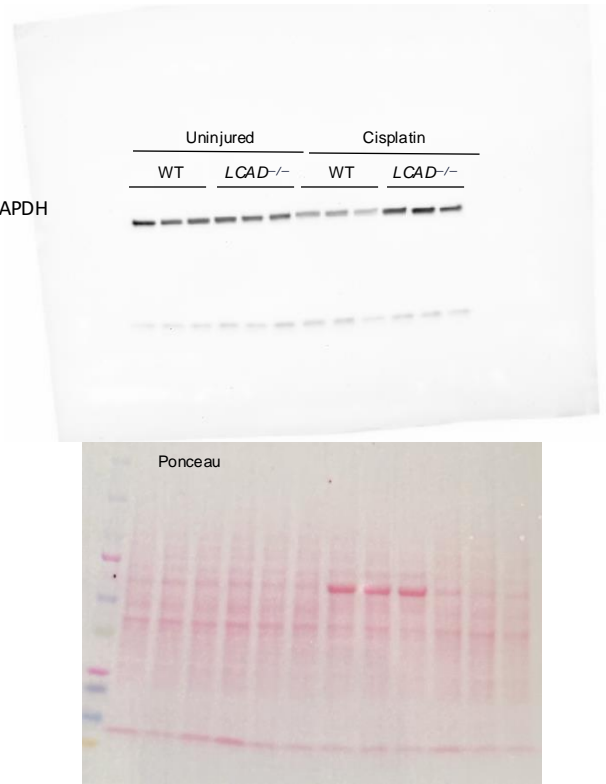

## Figure 8D (PEX5)

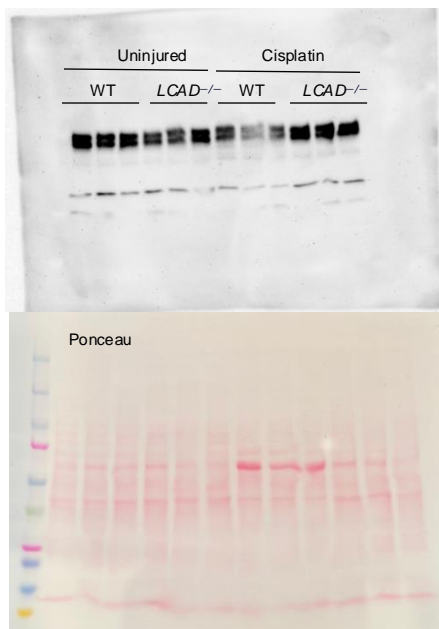

## Figure 9D (GPX4)

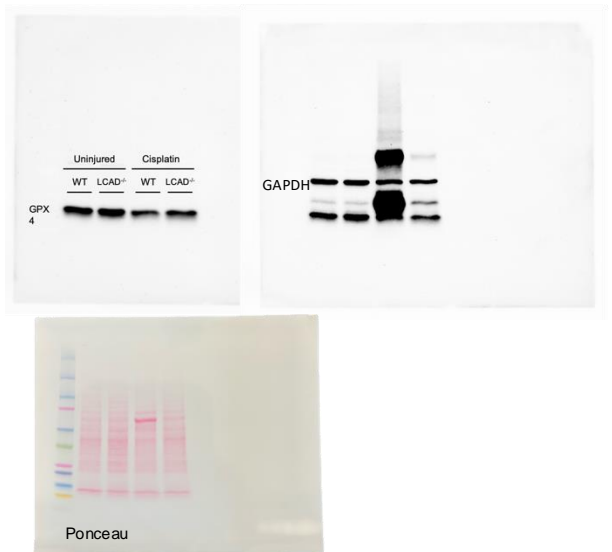

Supplement: Unedited blot and gel images [file jciinsight-10-186073-s326.pdf]
